# Values and preferences for hepatitis C self-testing among people who inject drugs in Kyrgyzstan

**DOI:** 10.1186/s12879-021-06332-z

**Published:** 2021-06-26

**Authors:** Guillermo Z. Martínez-Pérez, Danil S. Nikitin, Alla Bessonova, Emmanuel Fajardo, Sergei Bessonov, Sonjelle Shilton

**Affiliations:** 1 Department of Physiatrics and Nursing, University of Zaragoza, Calle Domingo Miral s/n, 50009 Zaragoza, Spain; 2 Global Research Institute Foundation (GLORI), 125 Suyumbaev Street apt 21, 720011 Bishkek, Kyrgyzstan; 3 Kyrgyz Network of Harm Reduction (KNHR), 21 Yug-2 Microdistrict apt 55, 720052 Bishkek, Kyrgyzstan; 4 Foundation for Innovative New Diagnostics, Chemin des Mines 9, 1202 Geneva, Switzerland

**Keywords:** Hepatitis C, Hepatitis C self-testing, Diagnostics, Values and preferences, Formative research

## Abstract

**Background:**

The prevalence of hepatitis C virus (HCV) among people who inject drugs (PWID) continues to be a major public-health burden in this highly stigmatised population. To halt transmission of HCV, rapid HCV self-testing kits represent an innovative approach that could enable PWID to know their HCV status and seek treatment. As no HCV test has yet been licenced for self-administration, it is crucial to obtain knowledge around the factors that may deter or foster delivery of HCV self-testing among PWID in resource-constrained countries.

**Methods:**

A qualitative study to assess values and preferences relating to HCV self-testing was conducted in mid-2020 among PWID in the Bishkek and Chui regions of Kyrgyzstan. Forty-seven PWID participated in 15 individual interviews, two group interviews (*n* = 12) and one participatory action-research session (*n* = 20). Responses were analysed using a thematic analysis approach with 4 predefined themes: awareness of HCV and current HCV testing experiences, and acceptability and service delivery preferences for HCV self-testing. Informants’ insights were analysed using a thematic analysis approach. This research received local ethics approval.

**Results:**

Awareness of HCV is low and currently PWID prefer community-based HCV testing due to stigma encountered in other healthcare settings. HCV self-testing would be accepted and appreciated by PWID. Acceptability may increase if HCV self-testing: was delivered in pharmacies or by harm reduction associations; was free of charge; was oral rather than blood-based; included instructions with images and clear information on the test’s accuracy; and was distributed alongside pre- and post-testing counselling with linkage to confirmatory testing support.

**Conclusions:**

HCV self-testing could increase awareness of and more frequent testing for HCV infection among PWID in Kyrgyzstan. It is recommended that peer-driven associations are involved in the delivery of any HCV self-testing. Furthermore, efforts should be maximised to end discrimination against PWID at the healthcare institutions responsible for confirmatory HCV testing and treatment provision.

## Background

Chronic hepatitis C virus (HCV) infection is a major public health problem [1]. The recent availability of direct-acting antivirals (DAA) has revolutionised the HCV treatment landscape, galvanising efforts to eliminate HCV by 2030 [2, 3]. To achieve HCV elimination, innovative approaches, such as decentralisation, integration and task-shifting of HCV services, are needed to facilitate linkage of infected individuals with each step of the cascade of care [4]. Patients and communities in many countries require safe and client-friendly HCV service-delivery models.

In 2016, just 20% of the HCV-infected population worldwide was aware of their HCV status [5]. Diagnosis rates are significantly lower in low- and middle-income countries (LMICs), with just 8% of people living with HCV diagnosed in 2016 [5]. The World Health Organization (WHO) testing guidelines for hepatitis B and C recommend routine inclusive testing approaches to ensure access to HCV testing for populations such as people who inject drugs (PWID), because of the stigma, discrimination, criminalisation and difficulties in accessing HCV prevention and care [6, 7]. In LMICs, point-of-care diagnostics are essential to reach people unaware of their HCV status [8]. Although the use of quality-assured, rapid HCV tests has expanded over recent years in LMICs [9], bridging the diagnostic gap will require additional strategies to reach communities who face challenges in accessing facility-based testing services. HCV self-tests are proposed as a screening approach to increase HCV diagnosis [10].

Since 2016, self-testing for HIV has been recommended by WHO as a strategy to increase individuals’ knowledge of their HIV status [11] and has been adopted in more than 70 countries [12]. Evidence suggests that HIV self-testing increases testing uptake and linkage to care [13, 14]. Lessons learned from HIV SELF-TESTING are encouraging with regards to the possibility of distributing HCV self-testing to highly affected communities. However, studies to assess the feasibility of HCV self-testing are crucial prior to its actual delivery in resource-constrained environments. A recent study among Vietnamese men who have sex with men (MSM) and PWID reported high HCV self-testing usability and inter-reader concordance, albeit performance errors were more frequent in the PWID group [15]. In China, a multicentre study reported high concordance between researcher-administered and participant-administered oral anti-HCV tests [16]. Good performance with self-administration of the OraQuick HCV Rapid Antibody Test was also reported in a study conducted in the United States [17].

In addition to usability and performance, a test’s acceptability to end-users must be explored. Some communities may be better empowered than others to demand HCV self-testing. PWID represent a priority population given the high burden of HCV in this group [18], although their rights to HCV care are frequently neglected [19, 20]. PWID participants in a qualitative study in London considered that HCV self-testing would be acceptable; however, it was also noted that barriers to accessing HCV treatment must be tackled for HCV self-testing to play a meaningful role in halting the spread of HCV among PWID [21].

Knowledge about HCV may be greater among PWID than the general population because many nongovernmental organisations (NGOs) have long integrated HCV prevention in their harm reduction programmes. This is the case in Kyrgyzstan, where HCV prevalence is around 3% in the general population [22] but between 17 and 60.4% among PWID [23]. Kyrgyz harm reduction associations refer PWID to government-run HCV testing services and provide counselling to HCV-affected PWID. In Kyrgyzstan, HIV self-testinghas been available to MSM and people living with HIV (PLHIV) since 2019 [24]. Considering the favourable environment for HIV self-testing, as well as the support networks that PWID in Kyrgyzstan have, this population may be ready for HCV self-testing. Therefore, we conducted a qualitative study with the aim of exploring values and preferences towards HCV self-testing among PWID in Kyrgyzstan.

## Methods

### Study design

This qualitative study was conducted in Bishkek, the capital of Kyrgyzstan, between August and October 2020. Data collection methods included individual interviews, group interviews and participatory action research (PAR). The research was conducted by the Kyrgyz Network of Harm Reduction (KNHR) under the auspices of a global assessment of HCV self-testing, led by the Foundation for Innovative New Diagnostics (FIND) in cooperation with the University of Zaragoza.

### Population and sampling

PWID, defined as individuals ‘who inject psychotropic (or psychoactive) substances for non-medical purposes’ (WHO, 2014), comprised the study population. PWID aged > 18 years, of any gender identity, who inhabited Bishkek or Chui oblasts, and were willing to provide informed consent were targeted as research informants.

One female and one male from KNHR led the sampling process, whereby all beneficiaries of KNHR-supported services (e.g. sterile-needle supply, condom distribution, legal and social counselling, and referral services for HIV and HCV testing) who met the inclusion criteria were purposively sampled. Potential informants were invited to participate in the study. Individuals who expressed an interest (*n* = 52) were invited to an individual, face-to-face explanation of the research aims and procedures. Two females and six males declined to participate.

Subsequently, 37 of the 44 individuals who did not decline to participate responded to a phone call from KNHR and consented to partake in an individual interview, a group interview or a PAR session. With the aid of the already recruited informants, an additional ten informants, who were not beneficiaries of KNHR-supported services, were recruited via snowball sampling techniques and invited to partake in the PAR session. Notably, non-beneficiaries might not have been recipients of KNHR services at the time of the study, but many were known to KNHR as they were considered suitable candidates to access and benefit from harm reduction services.

### Data collection and analysis

Participants’ written informed consent was obtained prior to data collection. All interviews were conducted in-person in Bishkek, using the Russian language. Fifteen individual interviews, one female-only group interview (*n* = 6) and one male-only group interview (*n* = 6) were conducted at the KNHR offices. Upon finalisation of the individual and group interviews, a PAR session was organised in a hotel in Bishkek (*n* = 20).

Individual and group interviews were led by a 42-item, semi-structured guide that aimed to explore four themes: knowledge of HCV; awareness of HCV testing; values around HCV self-testing; and preferences for HCV self-testing delivery to PWID. The interviews were audio recorded. Recordings were transcribed verbatim into a single question-by-question MS Excel® matrix.

**Fig. 1:**
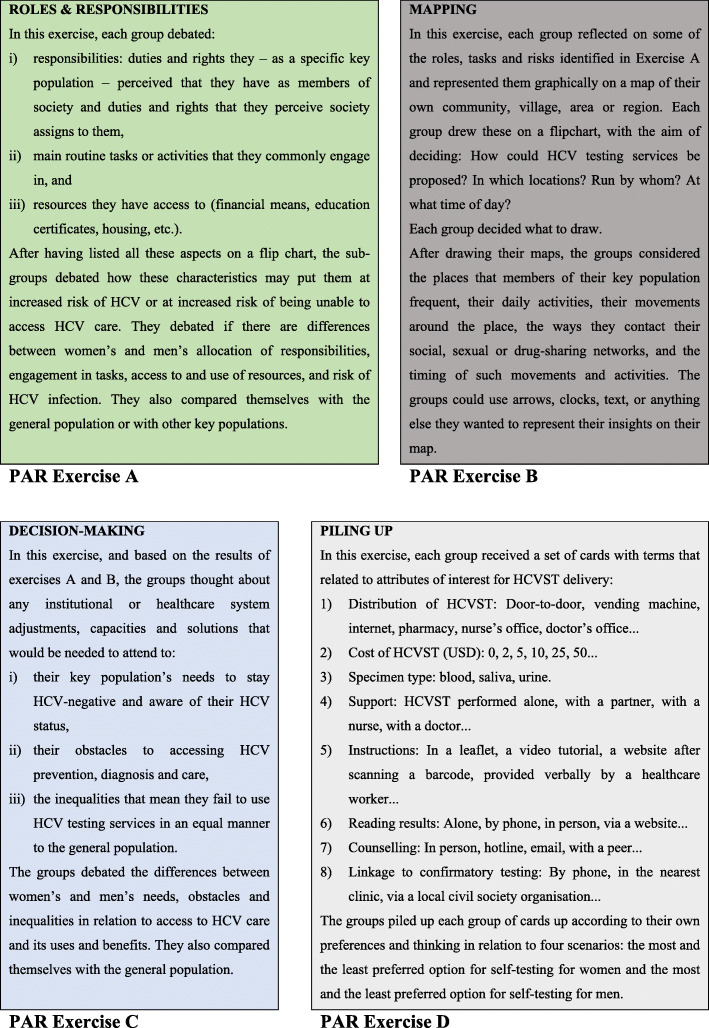
A description of the participatory action research (PAR) exercises

During the PAR session, the attendees were divided into four sub-groups: female-only KNHR beneficiaries (*n* = 5); female-only non-beneficiaries (*n* = 5); male-only KNHR beneficiaries (*n* = 5); and male-only non-beneficiaries (*n* = 5). Each sub-group was tasked with completing a set of four predefined exercises to explore the preferences of PWID for HCV self-testing delivery (Fig. 1). The exercises drew from previous community-grounded participatory planning experiences in resource-constrained settings [25]. The PAR session was not recorded. Photographs and scans were taken of the results of the four exercises. All results were compiled into a single MS Word® document for analysis.

Data collection, transcription and analysis of the MS Excel® and MS Word® datasets was performed contemporaneously. A thematic analysis, informed by Kielmann et al.’s guidance for non-social scientists engaged in the conduct of qualitative health research [25] was considered an appropriate methodological approach for analysing the data. Four-pronged scrutiny of the datasets was conducted. First, by reading, in chronological order, one interview record after the other. Second, by analysing the interviews in a question-by-question manner (i.e. 42 items). Third, by analysing the findings of the interviews in a theme-by-theme manner (i.e. knowledge, testing, values, preferences) and filtering by sex, education and area of residence. Fourth, the findings of the interviews were cross-compared with the data obtained in the PAR sessions.

### Trustworthiness

Various methods to demonstrate trustworthiness were used to ensure the credibility of the research findings. Triangulation was applied to choices on study population (maximum variation sampling in terms of age, gender identity, and area of residence), data collection methods (individual interviews, group interviews, PAR) and data analysis approaches (i.e. by the research team, external advisers, and seven informants in a final triangulation meeting).

Upon finalisation of each individual or group interview, the interviewers jointly analysed the transcripts to detect social desirability bias and any topics that might merit further consideration in future encounters. Data analysis was initiated while data collection was ongoing; therefore, peer checking of the preliminary findings from the individual interviews during the group interviews, and of the findings of the group interviews during the PAR session, was possible.

We ensured that COREQ (COnsolidated criteria for REporting Qualitative research) guidelines were considered [26], that deviant voices were selected, and that intersectionalities of drug-use with other variables of interest (e.g. sex, rural location, and use of harm reduction) were considered.

### Ethical considerations

This research was approved by the Committee on Bioethics, under the Global Research Institute in the Kyrgyz Republic (Ref. 011082020–1). All informants provided written informed consent. During the consent process, all informants were informed about the research aims; the organisations involved; the risks derived from their participation; their right to stop participation at any time; their right not to respond to any question they did not want to; and that no questions about their own drug-taking history would be asked during data collection. A unique identifier was created to link each consent form to the informant’s audio-recording. To further protect the informants’ anonymity, all audio recordings were deleted once the analysis was complete.

## Results

In total, 47 informants partook in 15 individual interviews (6 female, 9 male), one female-only group interview (*n* = 6), one male-only group interview (*n* = 6), and one PAR session (10 female, 10 male). The mean age of the interview and PAR informants was 34.7 and 35.3 years, respectively. There were 37 informants who were linked to KNHR services, while 44 participants reported living with HCV. There were 27 and 20 informants from the regions of Bishkek and Chui, respectively. Diverse ethnicities were reported, although all informants were Kyrgyzstan citizens. More than two thirds of informants [27] were in a relationship and cohabiting with their partners. Almost half of informants [21] reported having completed vocational education; in terms of religion, 24 and 9 informants reported being Muslim and Orthodox, respectively. Aggregate demographic data are shown in Table 1.

**Table 1:** Aggregate demographic data for the study informants

Demographic details	Female (***n*** = 22)	Male (***n*** = 25)
Age range (years)		
19–25	4	3
26–35	7	8
36–45	7	11
46–55	4	3
	Avg. 35.6	Avg. 34.4
Participants in		
Individual interviews	6	9
Group interviews	6	6
Participatory action research session	10	10
Area of residence		
Urban	12	15
Rural	10	10
Education		
Primary completed	1	1
Secondary completed	6	6
Vocational/College	7	14
University degree	8	4
Ethnicity		
German	–	2
Karachai	1	–
Kazakh	2	3
Korean	–	1
Kyrgyz	4	7
Russian	9	6
Tajik	–	1
Tartar	3	4
Ukrainian	1	–
Uzbek	2	1
Religion		
Muslim	10	14
Orthodox	6	3
Non-religious	6	8

### Knowledge of HCV

Awareness that HCV is a virus that can be transmitted via exposure to contaminated blood and sexual fluids is common among PWID. Fatigue, poor appetite and weight loss were identified as symptoms of hepatitis C in its earlier stages. Some informants mentioned that HCV is hard to detect as it does not initially manifest with acute symptoms; another noted that HCV infection is a ‘silent killer’ that can lead to cirrhosis and cancer:

*The disease can stay undetectable for years, it will be inside the body but there won’t be any symptoms, and yet the liver cells will be dying. First, it can convert into cirrhosis, and the second stage is the coma of the liver, which leads to death.*

(female, 35 y.o, Bishkek)

Awareness of the effectiveness of HCV therapies (i.e. sofosbuvir and daclatasvir) was limited. A few informants suggested that current treatments stop ‘multiplication’ of the virus but do not completely remove it from the body. Many informants noted that lack of knowledge about HCV treatments may be associated with the fact that healthcare workers refuse to treat PWID.

PWID who share syringes or other drug paraphernalia were described as being at risk of contracting HCV. Nevertheless, in the informants’ opinion, anyone could contract HCV following a blood transfusion or other medical procedure performed with non-sterile equipment. Despite these risks to the wider population, the general public’s attitudes towards people living with HCV were described as ‘negative’ and ‘suspicious’, or characterised by ‘fear’, ‘panic’, or ‘confusion’. To some, these attitudes correlated with ‘hostile’ attitudes towards PWID:

*Whenever I visit a lab to get some kind of a blood test, the nurses always ask me whether I am a drug user. When I say yes, they ask me to wait in a queue, and they service me only after they are done with all other visitors. They think that reduces the risk of spreading the viruses that I may have.*

(female, 35 y.o, Bishkek)

Negative attitudes may also relate to HCV being a disease that is perceived to be ‘fatal’ and associated with ‘socially disadvantaged’ and ‘marginalised’ people. Some informants highlighted that, although PLHIV experience much greater stigma, the general population may use the terms ‘plague’, ‘leprosy’, or ‘celestial punishment’ to describe people living with HCV and that people living with HCV are perceived by the general population to be ‘guilty’ and ‘deserving of their sufferings’. One PAR attendee suggested that Kyrgyzstan is transitioning to an ‘Islamic state, slowly but surely, and now there is no proper dialogue established between religious leaders who support stigma against key populations and activists who represent the HCV community’.

Fear of stigma was especially common among the informants who lived in rural areas; they expressed that it is very challenging for PWID to conceal their HCV status because community members pay special attention to their lifestyle. There were multiple mentions of actual incidents of discrimination that informants disclosed in relation to either their drug-use history or their HCV-infected status, for example:

*When I told doctors and beauty staff about my HCV-positive status, they used to [ask me to] clarify whether I am a drug user. It is like neglect: “Why should we care about [drug users’] health. They are the ones who are responsible for living risky lives”.*

(female, 27 y.o, Chui oblast)

### Awareness of HCV testing services

Poor health status, engaging in risky behaviour, and the need to provide an HCV-status certificate at job or visa applications were among the reasons identified that could make PWID demand testing in an HCV testing site, be it government-, private-, or civil society-run. The government-run clinics were described as the least client-friendly and most time-consuming. The private clinics were described as ‘unaffordable’, although the ‘quality of testing is high’. Sites managed by civil society organisations were described as the most PWID-friendly:

*There was the opportunity to get tested for free at the narcological centre. I remember that such an opportunity was available at the Republican Infectious Clinic when there was a project that covered the effort of specially designated doctors at this clinic. We called them “friendly doctors”.*

(male urban PWID, 37 y.o, Bishkek)

Lack of money, confidentiality issues, long turn-around-times for results, unawareness of available testing services, low perception of risk, and fear of stigma and discrimination from healthcare workers were mentioned as the main reasons PWID refuse testing. For rural PWID, transportation was the main barrier to travelling to Bishkek or larger towns, where most laboratories are located. Informants mentioned that some medical staff lacked the skills and patience to draw blood from PWID whose veins are damaged by *dimedrol* (i.e. diphenhydramine pills, powdered and mixed with heroin before injection). Additionally, twelve males with a history of incarceration explained that due to a lack of privacy in prison clinics, imprisoned PWID are discouraged from having an HCV test.

As per the informants’ narratives, when anybody from the general population wants an HCV test, they would have to go through ‘lots of registration procedures, get checked by dozens of doctors requiring informal payments’, and risk that ‘rude medical professionals neglect’ them, especially if they suspect they are a PWID or sex worker. Nevertheless, some informants claimed that barriers for HCV testing ‘are not that significant’ for PWID because HCV testing forms part of Kyrgyzstan’s national HIV strategy, targeting key populations. HCV testing is also routinely offered to pregnant women, although four female informants complained that it was ‘unfair’ to charge them for an HCV test during pregnancy as no clinical action would be taken because HCV treatment is, reportedly, denied to PWID.

### Value of HCV self-testing

Many informants reported previous use of self-tests for pregnancy, HIV, alcohol and drugs. There was general appreciation for allowing PWID to self-test for HCV. It was considered that HCV self-testing would increase its users’ confidentiality as PWID using HCV self-testing would not need to disclose sensitive information, could choose a safe location and convenient time to self-test, and could save time they would otherwise spend travelling and waiting for results.

*People who care about their privacy would love to know their diagnosis and get tested on business trips when travelling to another country so that people around do not know them in person. Having self-tests available would allow them to manage testing procedures at any time whenever it is convenient to them.*

(male, 49 y.o, Bishkek)

HCV self-testing would seemingly be the only HCV-testing opportunity for nine of the informants who lived in rural areas. Some mentioned that HCV self-testing would allow testing even in ‘COVID-19-specific lock-downs’ and that HCV self-testing would reduce the workload for laboratories. Importantly, HCV self-testing would help avoid interactions with ‘rude, discriminating’ healthcare personnel.

Perceived disadvantages of HCV self-testing were its anticipated unaffordable price, poor coordination between testing and treatment service providers, and difficulties with test usage and interpretation of results. All informants perceived oral self-tests to be easier to use than blood-based tests, although PWID might prefer the latter if they were more accurate, especially if they required capillary instead of venous blood.

*If it requires blood from veins, then it can appear problematic to me and my peers. We don’t have proper veins where blood can be taken from. I don’t know how this self-test will work. Maybe it would require just a blood drop from a finger, and then it would be acceptable and quite similar to saliva-based testing procedures.*

(male urban PWID, 26 y.o, Bishkek)

To some rural informants, urine-based HCV self-testing would be problematic considering the ‘traditional hut-like outdoor toilets’ in their villages and lack of ‘warm urban-style restrooms’ and ‘centralised sewer systems’. Nevertheless, the possibility of a pregnancy test-like urine-based HCV self-testing appealed to some female informants.

*That would be excellent. Take a kit, go to the restroom, pee and have a clear idea of the health status. You don’t have to go to a clinic or lab, sit in a queue in an overcrowded corridor, wait and communicate with medical staff.*

(female, 27 y.o, Chui oblast)

Almost all informants mentioned that the general population would be interested in HCV self-testing. The informants claimed that PLHIV, LGBT (lesbian, gay, transgender, bisexual) individuals, female sex workers and their ‘pimps’ and ‘clients, like truck drivers’*,* could also be interested. One female from Bishkek suggested that *‘*people like celebrities, actors, politicians, known by many people and wishing to preserve their privacy*’* could also be interested. Prison inmates could be interested, especially if HCV self-testing was distributed by agents external to the prison system:

*Such testing has to be available in prisons. There is a prison in the village where I live, so I do know how important it can be for those imprisoned.*

(male, 31 y.o, Chui oblast)

About one third of informants doubted HCV self-testing could help PWID access HCV treatment, as treatment is expensive in Kyrgyzstan even with imported generic drugs. Despite this anticipated barrier, most informants were aware that HIV-positive PWID would qualify for free HCV treatment:

*People with HIV are eligible for free HCV treatment. To qualify for this treatment, people have to submit evidence of Hep C. I know several people who are willing to first apply self-testing and only after they have a result showing their positive status, they are ready to apply to be included in this free treatment programme.*

(male, 44 y.o, Bishkek)

Informants considered that HCV self-testing would increase HCV testing rates. HCV self-testing would allow earlier detection of HCV, making treatment easier. Hence, HCV self-testing could, according to the majority of informants, help eliminate HCV in the future. HCV self-testing would provide awareness of their own health status and encourage them to avoid risky behaviour, use precautions, and seek advanced care. One male who regularly tested for HCV stated: ‘*I always pray: oh, let it be negative, and I give myself a word and swear to myself that in the future I will not do anything risky’.* Some informants suggested that even if HCV treatment were unavailable, there would opportunities to halt further HCV transmission if one knew they were HCV-positive:

*Whenever I go to a dentist or a nurse, I always warn them of my Hep C-positive status, and they encourage me to take advanced precautions with myself.*

(female, 35 y.o, Bishkek)

### Preferences for HCV self-testing

There was consensus that HCV self-testing should be available at any time during the week, ideally from pharmacies or NGOs. HCV self-testing could also be delivered to PWID via harm reduction services, infectious disease clinics, and prisons. Delivery of HCV self-testing in non-governmental peer-run shelters could be an option for LGBT and/or female sex worker PWID. The delivery of HCV self-testing via NGOs would be helpful in minimising illegal re-selling of HCV self-testing, as was reported with condoms and syringes. A male from a town where drug dealers were involved in a needle exchange programme noted that these individuals could become involved in HCV self-testing delivery:

*We would prefer to get tests through the outreach staff at the NGOs that we collaborate with. Let it be a part of needle-exchange services. Why not make them available through pharmacies? Maybe vending machines installed in the trade centres or near the pharmacies. I don’t know whether it would be all right to make them available through drug dealers... but why not?*

(male, 30 y.o, Chui oblast)

Although infectious disease specialists were recommended for HCV self-testing distribution, infectious disease clinics are only located in larger towns, their opening hours are inconvenient for many PWID, and they were not fully functioning during the COVID-19 lockdown. To overcome time and access limitations, vending machines and pharmacies would be acceptable because of their 24/7 availability. The vending machines should ‘not have a videocam installed’*.*

Receiving a brief HCV self-testing counselling session from a pharmacist rather than a doctor was thought to be quicker and cheaper. In the PAR session, a voucher-based distribution model for pharmacies was proposed, whereby PWID clients of harm-reduction NGOs receive ‘commodity vouchers’ they could exchange in a pharmacy for an HCV self-testing kit.

Regarding HCV self-testing provision in prison clinics, it was noted that HCV-infected prisoners would be afraid of violence following any unwanted disclosure of their HCV status by prison healthcare personnel. Four males with a history of incarceration suggested that client-friendly HCV self-testing protocols be implemented in prisons to ensure coercion-free HCV self-testing uptake among prisoners at risk of HCV. During the PAR session, female informants were supportive of rolling out HCV self-testing in all Kyrgyz prisons; however, the sub-group of male non-beneficiaries was concerned that the ‘whole prison culture discourages inmates from performing anything that requires privacy and confidentiality’. Privacy and confidentiality concerns were greater in rural areas; hence, rural informants were enthusiastic about the possibility of ordering HCV self-testing kits online or via a toll-free line.

Three rural informants mentioned that religious leaders could distribute HCV self-testing. Female beneficiaries attending the PAR session mentioned that neither religious leaders nor police officers should be involved in any provision of HCV self-testing information to PWID. One female explained why practising Muslims would accept HCV self-testing if it was recommended by religious figures:

*Mosques are the most visited public places in my village. Not schools, not clinics, not shops, but mosques. If religious figures like the mullah or the priest can be engaged… it would be great. Listen, just add a label with the word halal: it will attract thousands of Muslims to HCVT.*

(female, 41 y.o, Chui oblast)

Although this informant suggested that the word *halal* could be present on HCV self-testing kits, most informants opined that the packaging should be as neutral as possible, ‘as a household item’, with no mention of HCV.

*Imagine that you apply a self-test in the bathroom and then dispose of it as a part of regular trash. Everyone in the family will know that you were tested for Hep C and start asking questions... Maybe put a label on the pack that it has nothing to do with Hep C.*

(female, 37 y.o, Bishkek)

Providing information about a test’s accuracy was described as key to impact the decision of PWID to self-test, request confirmatory testing, or take a repeat self-test. If information on accuracy is unclear, some PWID may self-test several times to ensure result consistency. As one informant put it, ideally ‘only one test out of 1000 should show false results’.

HCV self-testing kit instructions should include detailed information on the test’s accuracy, images to guide its correct use, and steps necessary to request confirmatory testing. Gender-sensitive guidelines with images of women wearing scarves and use of the pronouns she/her instead of the usual he/his were proposed. Paper-based and web-based instructions should be in multiple languages. Large font sizes should be employed so that the elderly can read the instructions; PAR attendees noted that guidelines for blind people were also necessary. It was suggested that the instructions include a QR code so those with a smartphone could easily view an online video tutorial. It was emphasised that to facilitate home use of HCV self-testing and prevent any difficulties in manipulating the devices, PWID should be engaged in designing the packaging and guidelines.

*It’s important to show people who decide to apply self-tests, how to interpret the results. I remember tests for marijuana where users were quite confused with the stripes. If there are two stripes, was that the positive result or negative? I remember those marijuana tests did not have any inserts with guidelines, and no links to web-based resources. So, hopefully, the Hep C tests will be accompanied by that stuff.*

(female, 33 y.o, Chui oblast)

Informants mentioned that perceived ability to perform the test would be a key factor for PWID to decide on whether to test at home by themselves, with their friends or partners, or to request assistance from a healthcare worker. Although the majority affirmed that they would prefer to test alone, if assistance from a third party were necessary, they would feel most comfortable with a peer or a harm reduction site staff-member.

There was consensus that PWID would prefer to receive HCV self-testing for free. In the PAR session, 7.0 USD was proposed as the maximum amount that PWID may be willing to pay, should HCV self-testing not be provided for free in Kyrgyzstan.

‘Denial’, ‘fear’, ‘depression’, ‘panic’, ‘aggressiveness’, desire to ‘blame’ someone, and ‘suicide attempts’ were mentioned as possible reactions to a positive HCV self-testing. None of the few informants who mentioned suicide actually knew of any suicide attempts among their acquaintances following a positive infectious disease test result. To minimise the occurrence of these responses, it was emphasised that self-test users must be encouraged, either at point-of-delivery or in the kit’s instructions, to immediately contact an easy-to-reach trained person following a positive result.

Some mentioned that to prevent intimate-partner violence, the provision of pre-test couples’ counselling could be helpful. Reportedly, a positive HCV self-testing result could make some men suspect that their partner has a lover:

*Just imagine, a woman tests HCV-positive. But her spouse tests negative. A jealous guy can suspect her of being unfaithful and perform violence.*

(male, 48 y.o, Chui oblast)

NGO-led or *‘*peer-based escorting’ to confirmatory testing was proposed to overcome the challenges that PWID could face in linking to post-HCV self-testing care. To motivate PWID using HCV self-testing to request care, it was suggested that PWID with experience of confirmatory testing share their ‘success stories’ with the community. Some informants noted that to encourage confirmatory testing uptake among PWID afraid of HCV-related stigma, it would be preferable to test in ‘neutral territories’ that were not associated with HIV or drug treatment. Two informants mentioned that, rather than expecting PWID to travel to a clinic, paramedics could be invited to their trusted NGOs for blood collection and testing.

Concerns about the implications of post-HCV self-testing care were frequently expressed by all informants. Travelling to clinics, waiting in crowded corridors, incurring high expenditure, and having to interact with medical personnel were mentioned as deterrents for linkage. A common concern was the perception that doctors and nurses were ‘uneducated’ and ‘corrupt’ and suspected of breaching the confidentiality of their HIV/STI-infected clients. The difficulty for PWID to find a ‘qualified’ and ‘honest’ medical professional was expressed. HCV treatment provision to PWID following a confirmed HCV result would greatly depend, as per their narratives, on medical personnel’s commitment to caring for PWID ‘with dignity’.

## Discussion

This research contributes to the scientific evidence on the acceptability of HCV self-testing for populations that are at increased risk of HCV acquisition and disease due to the neglect of their rights to infection prevention and healthcare provision. PWID in Kyrgyzstan already benefit from education on HCV prevention offered to them by harm reduction associations. However, our research suggests that despite PWID having a high-level of awareness of HCV as a health hazard, the stigma and discrimination they endure in government-run HCV diagnostic and care sites makes them prone to unknowingly live with HCV. Thus, in Kyrgyzstan, PWID consider that having access to free-of-charge oral and/or blood-based HCV self-testing that is delivered alongside pre- and peri-testing counselling and linkage to confirmatory testing support is an acceptable strategy that could improve HCV-status awareness and opportunities to receive HCV treatment among PWID.

As HCV self-testing kits are not currently commercially available in Kyrgyzstan, our informants discussed their theoretical acceptability only. Nevertheless, their opinions were sufficient for this research to act as guidance when planning the delivery in the near future of HCV self-testing to PWID. For PWID to demand and use HCV self-testing, it should be free of charge or ≤ 7.00 USD; require an oral specimen unless the accuracy of an HCV self-testing requiring finger-prick blood is higher; be provided where PWID are treated with respect (e.g. pharmacies, NGOs); have packaging with no mention of HCV; and have clear instructions on how to request confirmatory testing following a positive result.

This guidance can be generalised to the implementation of HCV self-testing delivery beyond Kyrgyzstan. Similar aspects to those discussed by our informants were also mentioned by PWID discussing the acceptability of HCV self-testing among PWID in London [21] and were reported in HCV self-testing usability and acceptability studies conducted among PWID and MSM in Vietnam, Egypt, Georgia, China and Kenya [15, 28]. Furthermore, there are multiple similarities between narratives around HCV self-testing as expressed by Kyrgyz PWID and those around HIV self-testing expressed by key populations targeted in HIV self-testing research [29, 30]. For example, in terms of willingness-to-pay, our informants thought HCV self-testing should be offered freely or very cheaply to groups such as PWID, while the range of acceptable costs for HIV self-testing in LMICs ranged from < 1.00 to > 20.00 USD, based on 2015 exchange rates.

These similarities are a source of optimism, as they indicate that low-cost HCV self-testing kits could be as accepted and effectively used in real-world public health as HIV self-testing has been to date. However, HCV programmes are usually underfunded in comparison with HIV programmes. It must also be noted that the context and maturity of national HCV self-testing programmes is very different from the reach and maturity of HIV programmes since WHO first recommended HIV self-testing in 2015. Just a handful of countries have instituted national hepatitis C treatment programmes [27]. Research on the impact of HCV self-testing with a focus on linkage to care is needed. Linkage is especially important in Kyrgyzstan since, based on our informants narratives, the National HCV Program may only cover HCV treatment costs for PLHIV [31]. Hence, massive structural changes are essential to improve current HCV provision. Otherwise, even if HCV self-testing were successfully delivered in resource-constrained settings such as Kyrgyzstan, these successes could be short-lived should barriers to HCV-infected PWID receiving DAAs following a positive HCV self-testing result be perpetuated.

DAAs are neither affordable nor available for all individuals in many jurisdictions [27]. This is a deep-rooted inequality that must change to ensure acceptability of HCV self-testing among the most vulnerable groups. Another deep-rooted inequality to address is that standards for sensitive data protection are not guaranteed for stigmatised populations in many health jurisdictions worldwide. In this regard, and as in most studies on HIV self-testing acceptability, convenience and privacy were cited as advantages of HCV self-testing by our research informants. Huge efforts have been made in the past three decades to strive for zero stigma towards PLHIV. Both PWID and HCV-infected people have failed to see the same effort to promote zero stigma towards them. Fear of stigma and discrimination from healthcare workers and the general community following an HCV diagnosis must be considered by healthcare programme coordinators. Even if it were easy to perform HCV self-testing in private, if healthcare staff are not trained, respectful and culturally-sensitive individuals with non-judgemental attitudes and with a commitment to keep their HCV patients’ data confidential and their rights to care safeguarded, HCV self-testing would not be successful in any marginalised community, such as PWID in Kyrgyzstan.

Concerns about social harms that could arise from an HCV self-testing positive result (including fear of intimate-partner violence) mirror concerns that arose from research into the theoretical acceptability of HIV self-testing prior to their arrival on the diagnostics market [32]. In our research, a few participants suggested some female users of HCV self-testing may be at risk of intimate-partner violence. However, there are no data to suggest this risk materialised from HIV self-testing implementation programmes. A 2017 systematic review of HIV self-testing studies noted one instance of social harm; however, this stemmed from an individual participating in a study without the consent of their partner, so the social harm was not directly related to HIV self-testing [14].

As with HIV self-testing, HCV self-testing is intended as an additional testing modality to complement conventional facility-based HCV testing, by expanding access to testing to groups that are hard-to-reach or neglected by healthcare workers. Any potential scale-up of HCV self-testing must be considered within the context of existing laboratory capacity. Efforts should be made to strengthen testing for viraemia or viral load to facilitate post-HCV self-testing access to DAAs. Unlike HIV, there is no global funding body for HCV, so HCV self-testing could be integrated into existing international and domestic systems for HIV self-testing.

Our research had some limitations. First, KNHR recruited the informants. There is a possibility that pre-existing rapport with the researchers due to informants’ previous engagement in KNHR activities led to their involvement. However, it must be noted how difficult it is to reach and engage with PWID in this study setting. Hence, although the sample size was small, the voices heard in this research should be listened to by HCV self-testing programme planners. Another limitation is that 10/47 informants were not linked to harm reduction services and that 44/47 informants were living with HCV. Values and preferences of HCV self-testing from PWID that not linked to harm reduction and that are HCV negative may be different. Undoubtedly, once regulations are passed to allow HCV self-testing distribution, associations offering harm reduction will face fewer challenges when promoting HCV self-testing among linked compared with non-linked PWID. To ensure the equitable scale-up of HCV self-testing to all PWID, irrespective of the support services they receive, further research should be conducted into the values and preferences of non-linked PWID as potential end-users.

## Conclusions

The PWID we interviewed considered HCV self-testing to be a useful, convenient and private testing method that would allow PWID to know their HCV status, free of perceived stigmatising contact with healthcare providers. If HCV self-testing were free-of-charge, oral-based, and delivered alongside mechanisms for counselling and linkage, it represents an affordable, effective and culturally congruent strategy to improve HCV-status awareness among PWID. For sustainable HCV self-testing delivery programmes targeting PWID in resource-constrained settings, peer-driven associations should be involved in programme planning. Efforts should be maximised to end discrimination against PWID at healthcare institutions as well as inequities in access to confirmatory HCV testing and treatment.

## Data Availability

The datasets used and/or analysed during the current study available from the corresponding author on reasonable request.

## Abbreviations

COREQ: COnsolidated criteria for REporting Qualitative research
DAA: Direct-acting antiviral
FIND: Foundation for Innovative New Diagnostics
HCV: Hepatitis C virus
HIV: Human immunodeficiency virus
Kyrgyz: Harm Reduction Network Association
LGBT: Lesbian, gay, transgender, bisexual
LMIC: Low- and middle-income country
MSM: Men who have sex with men
NGO: Nongovernmental organisation
PAR: Participatory action research
PLHIV: People living with HIV
PWID: People who inject drugs
WHO: World Health Organization
